# Fucosylated α_1_-acid glycoprotein as a biomarker to predict prognosis following tumor immunotherapy of patients with lung cancer

**DOI:** 10.1038/s41598-019-51021-2

**Published:** 2019-10-10

**Authors:** Takehiko Yokobori, Shin Yazawa, Takayuki Asao, Nobuhiro Nakazawa, Akira Mogi, Rie Sano, Hiroyuki Kuwano, Kyoichi Kaira, Ken Shirabe

**Affiliations:** 1 Department of Innovative Cancer Immunotherapy, Graduate School of Medicine, Gunma University, Maebashi, Japan; 2 Department of General Surgical Science, Graduate School of Medicine, Gunma University, Maebashi, Japan; 3 Gunma University Initiative for Advanced Research (GIAR), Maebashi, Japan; 4 Department of Legal Medicine, Graduate School of Medicine, Gunma University, Maebashi, Japan; 5 Department of Respiratory Medicine, Comprehensive Cancer Center, International Medical Center, Saitama University Hospital, Hidaka, Japan

**Keywords:** Tumour biomarkers, Predictive markers, Predictive markers

## Abstract

Immunotherapy targeting immune checkpoint molecules has provided remarkable clinical benefits in cancer patients but no clinically relevant biomarker for predicting treatment outcomes exists. Recently, we demonstrated that glycan structures of serum α_1_-acid glycoprotein (AGP) changed dramatically in cancer patients and that α1,3fucosylated AGP (fAGP) levels increased along with disease progression and decreased responding to chemotherapy treatments. Here, the fAGP was analyzed in sera prospectively obtained from 39 patients with advanced lung cancer who underwent immunotherapy with anti-PD-1 antibody, nivolumab. Twenty-three patients had significantly high fAGP levels above the cut-off value (H-fAGP) at one month after starting the treatment and 20 patients in this group, whose tumor sizes did not decrease, maintained high fAGP levels continuously and subsequently died. However, the other 16 patients, whose fAGP levels decreased or maintained below the cut-off value (L-fAGP), survived during a 2-year observation even though 5 patients in this group had no tumor shrinkage. Accordingly, the overall survival rate was found to significantly correlate with the fAGP level. Multivariate analyses revealed that the H-fAGP was an independent risk factor for cancer progression. Therefore, the fAGP level appeared to be a reliable biomarker for predicting clinical efficacy of immunotherapy with nivolumab.

## Introduction

Inhibitory therapeutic strategies targeting immune checkpoints such as cytotoxic T lymphocyte antige-4 (CTLA-4)^1^, programmed cell death-1 (PD-1)^2^ and its ligand (PD-L1)^3^ have been proposed by their blockades with anti-CTLA-4^4^, anti-PD-1 and anti-PD-L1 antibodies^5,6^, respectively. Thereby, novel immunotherapies targeting patients’ lymphocyte receptors or their ligands have been approved to produce clinical benefits through enhancing their endogenous antitumor activities^7^. However, clinical responses to various immunotherapies are, in most of the cases, limited in a certain number of patients^6,8^. Further, treatments with such therapies are very high cost and involve severe immune-related adverse events in some patients^9,10^. Thus, establishment of a biomarker to predict treatment outcomes must be of clinical significance and an urgent need. To date, a series of candidates related to the immune checkpoint blockade^11–13^ as well as other molecules^14–16^ have been investigated, but no clinically applicable biomarker is present.

α_1_-Acid glycoprotein (AGP) is a major serum glycoprotein with a molecular weight of 41 to 43 kDa and with five *N*-linked, complex type glycan structures per one molecule^17^. Together with its potential physiologic significance as an acute-phase protein possessing diverse immunomodulating effects, a huge variety of structures synthesized in the molecule with large amounts of highly branched glycans have been investigated^18^. Hence, glycan structures in AGP have been demonstrated to change dramatically in a certain condition as well as during acute and chronic inflammation periods, in particular, in association with the presence of tumors^19,20^.

In our previous study^21^, a large numbers of serum samples from cancer patients were analyzed by a crossed affinoimmunoelectrophoresis (CAIE) with Con A lectin, *Aleuria aurantia* lectin (AAL) and anti-AGP antibody to determine the degree of branching and the extent of fucosylation in glycan structures of AGP. AGP glycoforms determined using the CAIE technique indicated that highly fucosylated tri- and tetraantennary glycan chains in AGP could predict patients with a good or poor prognosis. Whereas, enzymatic and molecular analyses of serum α1,3fucosyltransferase both in wild type and FUT6-deficient individuals together with those of fucosylation in AGP revealed that fucosylation of the AGP molecule was carried solely by the *FUT6* gene-encoded hepatic α1,3fucosyltransferase, and that no α1,6, α1,2 or α1,4 but only α1,3fucosylated linkage was present in the AGP molecule^22–24^. Furthermore, we developed a MALDI-TOF-MS system with a novel operation software (AGPAS) to analyze glycan structures of AGP comprehensively and determined a relative abundance of α1,3fucosylated glycans with tri- and tetraantennary glycan chains in AGP (FUCAGP)^25,26^. Accordingly, it was found, for the first time, that α1,3fucosylated AGP could be an appropriate marker of disease progression and prognosis in various cancer patients, and that abnormally high levels of FUCAGP were found in patients with esophagus, stomach, lung, breast, liver, pancreas, colon and rectum carcinomas who had a poor prognosis^25,26^. It was of particular interest that recurrence and/or metastasis occurred in patients who had been undergoing chemotherapies and/or radiation after operation was detected by elevated levels of FUCAGP in advance before their determinations with computerized tomography (CT) scans and diagnoses with conventional tumor markers.

Recently^27^, we have established an enzyme immunoassay (EIA) method to determine levels of α1,3fucosylated AGP (fAGP) by using anti-AGP antibody and AAL with additional endeavor to improve sample handling and antibody preparation. This simple antibody/lectin EIA allowed a rapid determination of the fAGP level in serum samples from patients under various chemotherapy treatments.

Here, we measured fAGP in serum samples from patients with advanced lung cancer who received immunotherapy with anti-PD-1 antibody, nivolumab after repeated treatments with unsuccessful chemotherapies, and evaluated the fAGP as a potential biomarker for treatment responses and outcomes.

## Results

### fAGP levels in serum samples from patients treated with nivolumab

**Figure 1:**
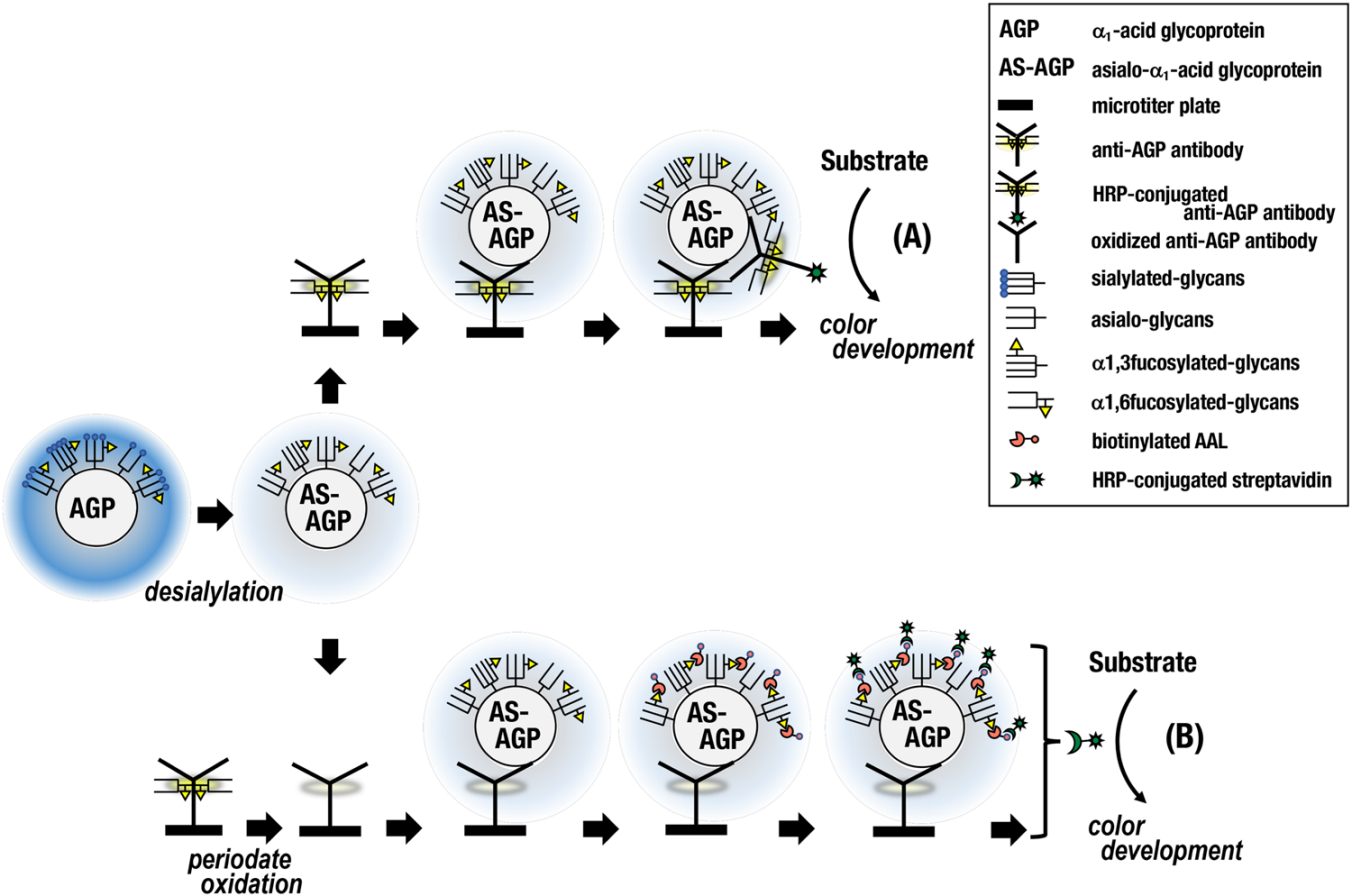
A schematic of a sandwich-type ELISA to determine levels of AGP (**A**) and an antibody/lectin EIA to determine levels of fucosylated AGP (**B**) in the same sample. Serum samples were treated with neuraminidase to prepare asialo (AS)-serum samples for both assays. Anti-AGP antibody coated on a 96-well microtiter plate for the EIA (**B**) was periodate oxidized to disrupt glycans including α1,6fucosylated ones attached.

**Figure 2:**
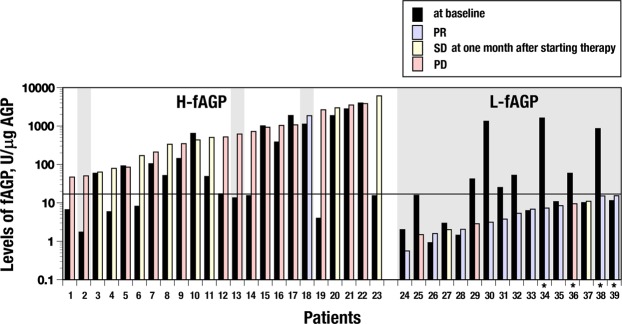
A comparison of the fucosylated AGP level between before and after administration of nivolumab. The fAGP level in each patient (n = 39) was measured before and after starting the administration of nivolumab. Accordingly, the fAGP level was measured at baseline and at 1 month in most of the patients and at 3 (No. 36) and 6 month (Nos 34, 38 and 39). The former was commonly indicated with a black-colored bar, and the latter was with differently-colored bars grouped according to the classification of the RECIST criteria^28^ conducted at the moment; PD (red), SD (yellow) and PR (blue), respectively. The solid line at 16.67 U/μg indicates the cut-off value. A gray-colored screen indicates patients who were alive during the observation time (see the details in Text).

Levels of fAGP in 39 patients treated with nivolumab were determined by a serial conduction of ELISA for AGP levels and then EIA for fAGP levels as the amount of α1,3fucosylated AGP per 1 μg of AGP (Fig. 1). Changes in the fAGP level determined at baseline and one month after starting nivolumab administration were compared in respective patients who are originally classified according to the RECIST criteria^28^. Accordingly, 12 patients showed partial response with decrease of their tumor sizes (PR). Ten patients whose tumor sizes did not change and 17 patients whose tumor sizes increased were classified as stable disease (SD) and progressive disease (PD), respectively. Twenty-three patients including 1 patient with PR, 8 patients with SD and 14 patients with PD showed high levels of the fAGP above the cut-off value at one month after starting the treatment (H-fAGP) (Fig. 2). Twenty patients whose tumor sizes did not decrease retained the level continuously and subsequently died. Further, in this group, two patients with PD (Nos 2 and 13) moved to the next line of treatment with chemotherapy after futile administration of nivolumab several times. They seemed to respond to the chemotherapy and survived during the observation time of this study. The patient (No. 18) was classified as PR due to the occurrence of tumor shrinkage at one month but then developed aspiration pneumonia and had a poor condition after 3 months with 7 times administrations accompanying discontinuous therapy and palliative care.

Whereas, the other 16 patients including 11 patients with PR, 2 patients with SD (Nos 27 and 37) and 3 patients with PD (Nos 25, 29 and 36) possessed low levels of the fAGP below the cut-off value (L-fAGP) even though 4 of them (Nos 34, 36, 38 and 39) showed delayed changes of the level (see below). All these patients with L-fAGP including 5 patients whose tumor sizes did not decrease showed a good prognosis and survived during a 2-year observation of this study.

It was therefore striking that classification groups of patients based on the RECIST criteria were not completely reflected in their clinical prognosis.

### Clinicopathological significance associated with fAGP levels

The relationship between clinical background of the patients and fAGP levels was investigated among two groups with L-fAGP (n = 16) and H-fAGP (n = 23). There was no significant correlation between the fAGP level and age, gender, smoking status, pathology, driver mutations including epidermal growth factor receptor (*EGFR*) and anaplastic lymphoma kinase (*ALK*) genes, or frequencies of pre-treatment (Table 1). However, there were statistically significant correlations between the fAGP level and immune-related adverse events (*P* = 0.028), frequencies of nivolumab treatment (*P* < 0.0001), clinical responses to nivolumab (*P* < 0.0001) and reasons for leading to treatment discontinuation (*P* = 0.0002).

**Table 1:** The relationship between clinical background of patients and levels of fucosylated AGP changed after administration of nivolumab.

Agents		Patients with		*P* value
		L-fAGP (n = 16)	H-fAGP (n = 23)	
Age (years)	mean ± S.D.	67.9 ± 7.8	64.3 ± 11.5	0.29
Gender	male	13	16	0.41
	female	3	7	
Smoking status	smoker	15	16	0.07
	nonsmoker	1	7	
Pathology	adenocarcinoma	14	17	0.3
	SQC	2	6	
Driver mutations	*EGFR*	1	4	0.54
	*ALK*	0	1	
	wild	12	13	
	unknown	3	5	
Frequencies of pre-treatment^a^	mean ± S.D.	2.65 ± 2.58	2.83 ± 1.65	0.77
Immune-related adverse events	absent	10	21	0.028
	present	6	2	
Frequencies of administration^b^	mean ± S.D	20.63 ± 14.04	5.22 ± 3.66	<0.0001
Clinical responses to nivolumab	PR	11	1	<0.0001
	SD	2	8	
	PD	3	14	
Reasons for leading to treatment discontinuation	disease progression	5	21	0.0002
	immune-related adverse events	4	0	
	aspiration pneumonia	0	1	
	death by AMI	0	1	
	treatment continuation	7	0	

SQC, squamous cell carcinoma; *EGFR*, epidermal growth factor receptor; *ALK*, anaplastic lymphoma kinase; AMI, acute myocardial infarction. ^a^chemotherapy. ^b^nivolumab.

### Changes in fAGP levels of patients with L-fAGP and H-fAGP

**Figure 3:**
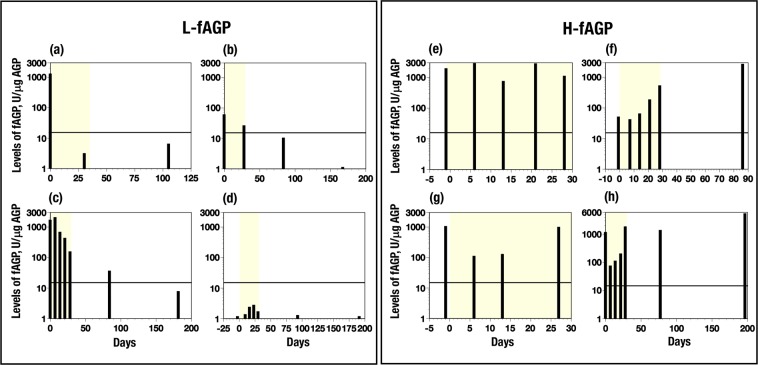
Changes of fucosylated AGP levels in serum samples from patients treated with nivolumab. L-fAGP contains patients whose levels decreased below the cut-off value within one month (**a**) (No. 30) and a few months (**b**) (No. 36) and (**c**) (No. 34), and whose levels remained below the cut-off value (**d**) (No. 26). H-fAGP contains patients whose levels retained high (**e**) (No. 17), increased gradually within one month (**f**) (No. 11) and showed transiently decreased levels during one month (**g**) (No. 15) and (**h**) (No. 18). Numbers noted in brackets are patients’ numbers in Fig. 2. The solid line at 16.67 U/μg indicates the cut-off value in this study. fAGP, fucosylated AGP. A yellow-colored screen in each group indicates a period of one month after starting administration (see the details in Text).

The fAGP level in each patient was measured at for a maximum of 6 month after starting the treatment to see changes in their levels. In most of the patients with L-fAGP, a decrease to below the cut-off value shortly after starting the nivolumab therapy was observed, which was sustained during the treatments (Fig. 3a). Four patients (Nos 34, 36, 38 and 39 in Fig. 2) showed delayed responses to reach below the cut-off value within a few months after starting the treatment (Fig. 3b,c). Further, 4 other patients (Nos 24, 26 to 28 in Fig. 2) showed very low fAGP level during the observation time (Fig. 3d). Lethal mutation of the *FUT6* genes which induces absence of fAGP in serum^23,24^ was not found in these patients, but 3 patients (Nos 24, 26 and 27 in Fig. 2) possessed only one mutated *FUT6* allele heterozygously. While, fAGP levels in patients with H-fAGP changed in 3 different profiles described below, but never decreased to below the cut-off value. *First*, the very high levels were sustained during the observation time (Fig. 3e); *second*, the high level continued to increase (Fig. 3f) and *third*, elevated fAGP levels decreased soon after starting the treatment, reflecting respond to the treatment, but after a short time, increased again (Fig. 3g,h).

### Changes in glycan chain structures of AGP following nivolumab therapy

**Figure 4:**
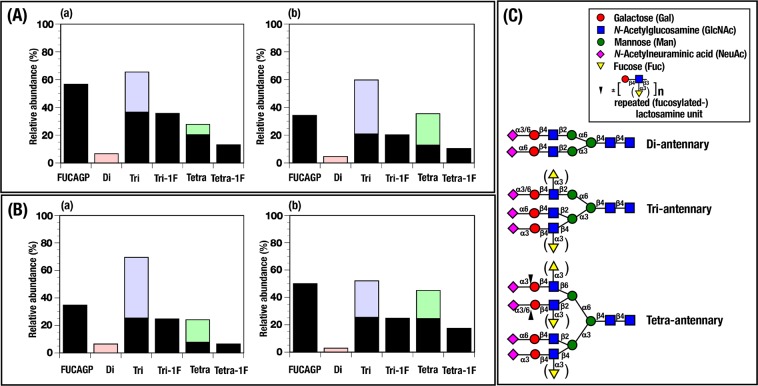
Changes of relative abundance of glycan chains in AGP from patients treated with nivolumab and symbolic representation of glycan chains present in AGP. Relative abundance of each glycan (%) was analyzed with a mass spectrometer and unfucosylated (open bar) and fucosylated (black-colored closed bar) glycans in AGP were calculated. Relative abundance of fucosylated glycans in AGP (FUCAGP) was obtained by adding fucosylated tri- and tetraantennary glycans. (**A**) fAGP levels of the patient No. 34 in Fig. 2 decreased from 1617.56 U/μg (a, at baseline) to 34.92 U/μg (b, at 3 month). (**B**) fAGP levels of the patient No. 11 in Fig. 2 elevated from 48.63 U/μg (a, at baseline) to 2590.9 U/μg (b, at 3 month). Di, di-antennary; Tri, tri-antennary; Tetra, tetra-antennary glycan chains. 1F, monofucosylated glycans.

In order to analyze detailed glycan chains of AGP in patients who received immunotherapy with nivolumab and to compare two different values indicating fucosylated levels of serum AGP which were determined as fucosylated AGP in serum (fAGP) and fucosylated glycans in serum AGP (FUCAGP), respectively, FUCAGP in patients with L-fAGP and H-fAGP was also analyzed by a mass spectrometer (Fig. 4A,B). As shown in Fig. 4C, AGP glycans consist of five complex type *N*-linked glycans including di-, tri- and tetraantennary glycan chains. All the fucosylated glycans found in AGP form the (sialyl) Le^X^ ((NeuAcα2,3)Galβ1,4[Fucα1,3]GlcNAc) determinant on the lactosamine (Galβ1,4GlcNAc) structure in both tri- and tetraantennary glycan chains including elongated tetraantennary ones with repeating lactosamine structures but not in diantennary glycan chain^25,26^. FUCAGP (%) of the patient with L-fAGP (No. 34) whose fAGP levels (U/μg) were 1617.56 at baseline and 34.92 at 3 month, decreased correspondingly from 56.7 to 34.27, respectively (Fig. 4A). Whereas, FUCAGP of the patient with H-fAGP (No. 11) whose fAGP levels were 48.63 at baseline and 2590.9 at 3 month, increased from 34.79 to 50.07, respectively (Fig. 4B). It was also demonstrated that FUCAGP determined in both patients with L- and H-fAGP before (Fig. 4a) and after (Fig. 4b) treatments consisted dominantly of mono-fucosylated glycans attached to tri- and tetraantennary glycan chains but few di-, tri-, tetra- or more-fucosylated glycans on the same glycan chains were present. No significant difference was observed in fucosylated levels of AGP glycans changed individually during the follows-up of the same patients (n = 10) when they were measure by either fAGP or FUCAGP (data not shown).

### The overall survival rate in patients treated with nivolumab

**Figure 5:**
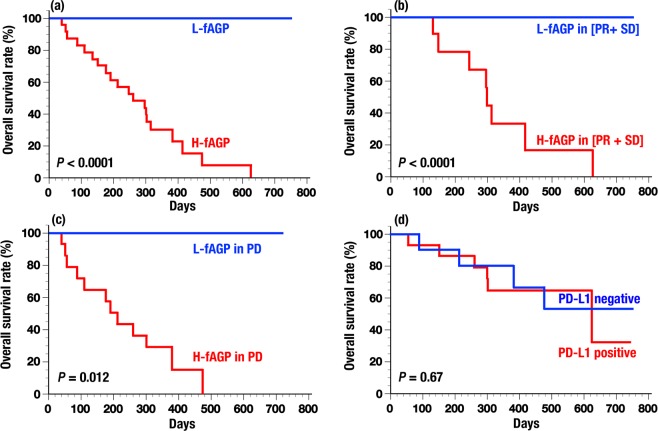
Overall survival rates of patients treated with nivolumab. Survival rates were compared alternatively between patients with L-fAGP (n = 16) and H-fAGP (n = 23) (**a**), patients with L-fAGP in PR plus SD (n = 13) and H-fAGP in PR plus SD (n = 9) (**b**), patients with L-fAGP in PD (n = 3) and H-fGAP in PD (n = 14) (**c**) and patients with PD-L1 negative (n = 10) and positive (n = 15) (**d**), respectively. See the details in Text.

The overall survival rate in patients was determined according to the fAGP level and other conventional predictive factors. When the survival rate of patients was compared between L-fAGP and H-fAGP, significant differences were observed in all patients (Fig. 5a, log rank *P* < 0.0001). Since 5 patients with SD and/or PD whose tumor sizes did not decreased but had L-fAGP were found to have a good prognosis (Fig. 2), the overall survival rate was also analyzed in patients with L- and H-fAGP both in PR plus SD whose tumor sizes did not increased and in PD whose tumor sizes increased, respectively. Significant differences were also observed in these subgroups when compared between L-fAGP and H-fAGP in patients with PR plus SD (Fig. 5b, log rank *P* < 0.0001) and in patients with PD (Fig. 5c, log rank *P* = 0.012). Additionally, the survival rate was evaluated in patients with the expression levels of PD-L1 (Fig. 5d, log rank *P* = 0.67) or tumor markers including CEA and SCC (log rank *P* = 0.089, figure not shown), but no significant difference was found.

### Univariate and multivariate analyses of predictive factors of cancer progression following nivolumab therapy

We undertook further evaluation of 7 agents including the fAGP level as predictive factors of cancer progression (Table 2). Both univariate and multivariate analyses indicated that the fAGP level was the only independent risk factor, predicting cancer progression.

**Table 2:** Univariate and multivariate analyses of the predictive factors related to efficacy of immunotherapy treatment.

Agents	Univariate analysis			Multivariate analysis		
	OR	95% CI	*P* value	OR	95% CI	*P* value
Age (≤65/>65) (yr.)	0.64	0.17–5.78	0.5	—	—	—
Gender (male/female)	1.42	0.33–6.19	0.64	—	—	—
Smoking (non-smoking/smoking)	0.38	0.07–1.83	0.23	—	—	—
Histology (adeno ca./SQC)	0.73	0.13–3.51	0.7	—	—	—
PD-L1 expression (negative/positive)	0.21	0.03–1.12	0.07	0.1	0.005–0.82	0.03
Levels of tumor markers (low/high)	2	0.41–11.45	0.39	—	—	—
Levels of fAGP (L-fAGP/H-fAGP)	6.74	1.63–35.9	0.0073	12.8	1.71–270.9	0.011

OR, odds ratio; Cl, confidence interval; adeno ca., adenocarcinoma; SQC, squamous cell carcinoma.

Levels of tumor markers were determined by concentrations of CEA and/or SCC in serum.

## Discussion

Through analyses of large numbers of serum samples from cancer patients and their follow-ups for a long period of time by means of CAIE and MALDI-TOF-MS^21,25,26^, we demonstrated previously that α1,3fucosylated glycans in AGP increased in association with tumorigenicity and could be a relevant biomarker for following patients who received treatments with various chemotherapies and/or radiation. Further, recently we developed an antibody/lectin EIA^27^, which seemed to provide a more simple and convenient method to measure fAGP as an alternative to α1,3fucosylated glycans in AGP^21^ and FUCAGP^25,26^. With this knowledge in hand, in the present study, we measured the fAGP level in 39 patients with advanced lung cancer who received immunotherapy with nivolumab. By measuring the fAGP level before and after treatment, we found that 20 patients with H-fAGP sustained high fAGP levels without possessing L-fAGP during the observation time and subsequently died. However, 16 patients showed to drop their levels below the cut-off value (n = 9) or to retain L-fAGP (n = 7) after treatment and had a good prognosis. It was of particular interest that such a significant drop of the fAGP level occurred after several times of the treatment even in patients whose tumor sizes increased or did not decrease at one month, and resulted in a good prognosis. Therefore, the fAGP level in 36 out of 39 patients measured in this study seemed to predict a good and/or poor prognosis compatibly in an early period. In addition, the rest of the 3 patients with H-fAGP who had discontinuation of the therapy due to aspiration pneumonia (No. 18) or progression of disease (Nos 2 and 13) were alive during the 2-year observation time with palliative care or the next line treatment of chemotherapy. Perhaps, in these 3 patients, a high fAGP level did not reflect cancer progression *per se*.

Hitherto, the clinical assessment of response to therapeutic interventions in patients with solid tumors has been conducted mainly based on the RECIST criteria^28^ assumed to detect early effects of cancer therapies. Whereas, the present results indicate that immunotherapy with the aid of immune checkpoint blockade redounds to some patients not in consistent with the RECIST criteria. It was pointed out recently that 10 to 20% of patients who had received immunotherapy treated with anti-CTLA-4 antibody showed increases of their tumor sizes at 3 months after starting the administration but no more tumor progression occurred without any additional intervention^9^. Thus, it must be possible that determination of fAGP level might advance accurate diagnosis of better treatment outcomes of patients.

MALDI-TOF-MS analyses of *N*-glycans in AGP concurrently conducted in this study revealed the structures of fucosylated glycans and their glycan chains in each AGP molecule. It was notable that monofucosylated tri- and tetraantennary glycans were predominantly found in fucosylated glycans in AGP which had a major influence on the fAGP level during the immunotherapy treatment. Interestingly, di- or trifucosylated glycans on the same glycan chains did not detected in patients who had received immunotherapy with nivolumab even though such fucosylated glycans were present in patients who had received various chemotherapy treatments as reported previously^23^.

Currently, biomarkers for cancer immunotherapy based on the immune checkpoint blockade have been investigated mainly to predict response to the therapy and occurrence of adverse effect using samples from patients’ tissues and/or blood^29^. Since immunotherapy with anti-PD-1 antibody targets PD-1/PD-L1 immune checkpoint blockade, the expression levels of related molecules such as the PD-L1 on tumor cells have attracted a great deal of attention for predicting response and efficacy to the treatment^5,30–32^. However, different results have been reported^33^, and in this study, no significant difference in the overall survival of patients with PD-L1 or tumor marker expression was found. However, in this cohort of patients, there were significant correlations between the fAGP level and the patients’ demographic variables including immune-related adverse events, frequencies of nivolumab administration, clinical responses to nivolumab and treatment discontinuation. It was also clearly observed that significant differences in the overall survival rate were present between patients with L-fAGP and H-fAGP beyond the RECIST criteria.

Furthermore, both univariate and multivariate analyses revealed that the fAGP level was an independent predictor of cancer progression in patients treated with nivolumab.

AGP has been recognized as an acute phase protein and an inflammation marker^17^. Further, it has been demonstrated that glycan structures as well as serum levels of AGP dramatically change during inflammation and that changes in the degree of branching and in the extent of fucosylation occur which discern acute and chronic inflammation^34–36^. It was more noteworthy that α1,3fucosylation on tri- and tetraantennary glycan chains occurred specifically along with inflammation and various clinical events including malignant transformation in various types of cancer as demonstrated in our previous studies^21,25–27^. Even though its exact physiological or pathological functions are currently unclear, immunomodulatory functions of AGP have been described together with possible involvement of certain glycan structures, in particular, α1,3fucosylated glycans^34,37,38^. One of the most interesting aspects of these assertions appears to be that the secreted α1,3fucosylated AGP molecules have a primary role in the feedback-response mechanism to suppress excessive inflammation (self-limiting), which might proceed with possible involvement of a cytokine network^18,35^. It was also strongly suggested from both previous and present studies that fAGP was synthesized rapidly, reflecting patients’ responses to treatment. It remains to be seen whether or not the fAGP could be validated clinically by using an increased number of samples across tumor types as a predictive biomarker for tumor immunotherapy together with analysis of detailed mechanisms to regulate fucosylation occurred in the AGP molecule during the therapy.

In conclusion, the fAGP which had been demonstrated previously to be a promising diagnostic and prognostic tumor marker, appeared to be a reliable biomarker for predicting clinical efficacy of immunotherapy with nivolumab as well as predicting long-term outcome.

## Methods

### Materials

Peroxidase conjugated ABC reagent, Vectastain Elite ABC standard kit was from Vecter Laboratories, Inc. (Burlingame, CA, USA). Antihuman AGP rabbit serum and Protein Block Serum-Free Reagent were from Dako (Carpinteria, CA, USA). Peroxidase conjugated anti-human AGP and Universal HIER antigen retrieval reagent were from Abcam (Cambridge, UK). KPL SureBlue TMB Microwell Peroxidase Substrate was from Sera Care Life Sciences (Milford, MA, USA). Anti-PD-L1 and SignalStain Boost IHC Detection Reagent were obtained from Cell Signaling Technology (Danvers, MA, USA). N-Histofine High Stain HRP (Multi) was from Nichirei Biosciences Inc. (Tokyo, Japan). PNGase F was from Roche Applied Science (Indianapolis, USA). BlotGlyco was obtained from Sumitomo Bakelite, Co. (Tokyo, Japan). Neuraminidase (*Arthrobacter ureafaciens*, 1U/ml) was purchased from Nacalai Tesque (Kyoto, Japan). Biotinylated *Aleuria aurantia* lectin (AAL) was kindly provided by Prof. Naohisa Kochibe, Gunma University. Tumor-associated antigens in serum samples measured in this study were carcinoembryonic antigen (CEA) and squamous cell carcinoma (SCC). Levels of each antigen were determined by an ELISA using the Cobas system (Roche for CEA) and the ARCHITECT system (Abbot for SCC) and standard cut-off values were set at 5.0 ng/ml for CEA and 1.5 ng/ml for SCC, respectively.

### Serum samples and ethical considerations

Serum samples were prospectively obtained from 39 patients with advanced lung cancer admitted to the Gunma University Hospital (Maebashi, Japan) and Hidaka Hospital (Takasaki, Japan) with strict adherence to the set guidelines for informed consent together with approval from the Ethics Committee of Gunma University Graduate School of Medicine and Hidaka Hospital. Additionally, approval was obtained from the Gunma University Ethical Review Board for Medical Research Test Samples from Human Subjects (Approval No. 1404). The later was intention to undertake clinical research and evaluate the efficacy of the anti-PD-1 antibody, nivolumab following unsuccessful chemotherapy. All experiments were performed in accordance with the relevant guidelines and regulations.

### Assessment of treatment efficacy

The clinical response to the treatment with nivolumab was assessed by using the ^18^F-FDG uptake and CT scans conducted at one month after starting administration^39^, and classified according to the Response Evaluation Criteria in Solid Tumors (RECIST)^28^.

### Determination of AGP and fAGP levels

Serum samples were collected basically at every week during 1 month, at every 3 month and for maximum of 6 month after starting the treatment. Serum samples collected were treated with sialidase (*Arthrobacter ureafaciens*, Nacalai Tesque, Kyoto, Japan) to prepare asialo-serum samples as described previously^27^. AGP and fAGP levels were measured by means of a sandwich-type ELISA and an EIA with periodate oxidized anti-AGP antibody and biotinylated AAL, respectively using asialo-serum samples (Fig. 1). Standard asialo-AGP for both ELISA and EIA was also prepared and used to make standard curves for determining levels of AGP and α1,3fucosylated AGP, respectively^27^. The amount of fAGP was ultimately expressed in terms of per one μg asialo-AGP as an arbitrary unit (U/μg). The cut-off value was set at 16.67 U/μg (=mean ± 2 S.D. value from healthy controls).

### Determination of glycan structures in AGP by a mass spectrometer

AGP preparations were purified from serum samples (n = 10) by an AGP-prep-DOCK^26^. *N*-Glycans of AGP and labeled *N*-glycans of AGP were prepared and purified as described previously^26^ using PNGase F (Roche Applied Science, Indianapolis, IN) and BlotGlyco (Sumitomo Bakelite, Co., Tokyo, Japan). Mass spectrometric data were obtained using a MALDI-TOF/TOF ultrafleXtreme (Bruker) and all the primary structures of AGP glycans were assigned with the aid of the AGPAS software^25,26^. The relative abundance of α1,3fucosylated tri- and tetraantennary glycans in AGP was defined as FUCAGP^26^.

### Sequence of the *FUT6* gene

Genomic DNA was extracted from peripheral blood samples by the standard phenol-chloroform extraction method. The *FUT6* gene locus was PCR-amplified as described previously^24^. Nucleotide sequences of the PCR products were determined by a direct sequence using specific primers of the target. The PCR product was also ligated into the pUC118 vector followed by transformation and cloning. Hence, 12 clones per sample were subjected to colony direct PCR, followed by sequence determination.

### Immunohistochemistry

Immunohistochemical staining of PD-L1 with anti-PD-L1 antibody (Cell Signaling Technology, MA) in each section obtained from patients was done according to the procedure described previously^39^. A semi-quantitative proportion scoring method was used to evaluate the positivity of tumors and each score was graded as follows; 1 = < 1%, 2 = 1–5%, 3 = 5–10%, 4 = 25–50% and 5 = > 50% of tumor cells were stained with the antibody. The section indicating scores with more than 3 was defined as positive in this study.

### Statistical analysis

The relationship between clinicopathological features and fAGP levels was investigated by using the Mann-Whitney and the Chi-squared tests. The Kaplan-Meier estimator graphs were generated for the overall survival and statistical significance was determined by using the log-rank test. Univariate analysis was done for each predictive factor together with stepwise multivariate analysis by using a logistic regression analysis model. For predicting cancer progression, the Akaike information criterion^40^ was applied to the statistically significance levels obtained in univariate analysis. All statistical analyses were undertaken by using the JMP software (SAS Institute Inc., Cary, NC).

## Data Availability

The datasets generated during and/or analyzed during the current study are available from the corresponding author on reasonable request.
